# Pea–cucumber crop rotation suppresses *Fusarium* pathogens by reshaping soil microbial communities and enhancing nutrient availability

**DOI:** 10.3389/fmicb.2025.1697343

**Published:** 2025-11-12

**Authors:** Jun Xu, Yuan Yao, Liu Pan, Ningyuan Zhang, Donghao Li, Xuehao Chen

**Affiliations:** 1 School of Horticulture and Landscape, Yangzhou University, Yangzhou, Jiangsu, China; 2 Tibet Academy of Forest Trees, Lasa, China

**Keywords:** cucumber *Fusarium* wilt, pea–cucumber crop rotation model, microbial diversity, soil nutrients, cucumber production

## Abstract

**Introduction:**

Pea–cucumber rotation combined with straw return as green manure is an environmentally friendly management strategy to suppress cucumber (*Cucumis sativus* L.) *Fusarium* wilt (FW) and alleviate continuous cropping obstacles.

**Methods:**

We evaluated the variations in soil microbial compositions and nutrient levels between long-term cucumber monocropping and pea–cucumber rotation patterns via metagenomic sequencing and determination of soil properties.

**Results:**

The study found that the bacterial communities exhibited marked diversity, whereas the *α*-diversity of fungal communities was significantly reduced. Based on the relative abundance of differential fungi and bacteria at the genus level, the genus *Bacillus* showed the highest abundance, with a two-fold increase, whereas *Fusarium* species exhibited a 4.9-fold reduction following the pea–cucumber rotation. Additionally, the contents of available nitrogen, potassium, and phosphorus in the soil increased by more than 1.3-fold after the rotation. Correlation analysis revealed that the genus *Bacillus* and available potassium were significantly and negatively correlated with *Fusarium* pathogens. Notably, the isolated *B. pumilus* and *B. safensis* strains significantly suppressed the growth of cucumber FW pathogens.

**Discussion:**

These findings provide valuable insights for optimizing the combination of soil *Bacillus* populations and nutrient availability to maintain soil ecosystem health and improve cucumber growth and yield.

## 1 Introduction

Continuous cropping in agriculture often leads to soil sickness, which is primarily caused by the accumulation of harmful pathogens or toxic substances, a decline in key microbial taxa, and reduced fertility in the crop rhizosphere, ultimately resulting in decreased crop yield and quality (Banerjee et al., 2019; Ma H. et al., 2025; Ma Z. et al., 2025). In particular, the long-term practice of continuous monocropping for many crops has a negative impact on soil health (Sun et al., 2021). China is widely recognized as the largest producer and consumer of greenhouse vegetables, with cucumber (*Cucumis sativus* L.) being one of the most economically important varieties worldwide (Feng et al., 2020; Yuan and Zhang, 2021; Zhang et al., 2024; Xin et al., 2025; Nie et al., 2025). However, continuous cropping obstacles in cucumber cultivation are mainly associated with the accumulation of *Fusarium oxysporum* pathogens and root-knot nematodes, which greatly hinder sustainable cucumber production.

Various approaches have been employed to prevent soil sickness, including chemical inducers (Bai et al., 2020), biofertilizers (Aloo et al., 2022), microbial inoculants (Chang et al., 2022; Giovanardi et al., 2025), green manuring (Jin et al., 2019a), and soil fumigation (Tong et al., 2025). However, crop rotation remains an ancient and well-established agricultural practice that can reshape soil microbial communities, enhance associated beneficial microorganisms, and improve soil fertility to overcome soil sickness in continuously cropped fields, thereby promoting agroecosystem sustainability and productivity (Wu J. et al., 2025; Wu H. et al., 2025). Continuous cucumber cropping has been reported to decrease yield and quality (Sun et al., 2021; Chen et al., 2022), and soil under different cropping systems has been extensively investigated in intensive greenhouse regions. For example, green garlic–cucumber rotation can regulate soil physicochemical properties and microbial community dynamics, thereby improving the soil environment and cucumber productivity (Ding et al., 2020). Similarly, cress–cucumber rotation alters the diversity, structure, and composition of soil microbial communities, which exerts positive effects on cucumber growth and development (Gong et al., 2021). Pepper–cucumber rotation also restructures rhizobacterial communities, facilitating the colonization of the cucumber rhizosphere by two *Pseudarthrobacter oxydans* strains (RH60 and RH97) through the enrichment of palmitic acid in pepper root exudates, thereby alleviating cucumber root-knot nematode infection (Tian et al., 2025). Overall, the soil microbial environment exerts a crucial influence on below-ground processes and crop performance. Nevertheless, although crop rotation practices can foster beneficial microbial communities that decrease pest pressure, suppress pathogens, and improve plant health, further in-depth studies are still required to elucidate these mechanisms.

Accumulating evidence has shown that pea (*Pisum sativum* L.) rotation or direct application as green manure can enhance soil nutrient content and uptake, including nitrogen levels, water retention, and overall system productivity (Gan et al., 2015; Chaudhari et al., 2020; Mesfin et al., 2023). Notably, this practice also stimulates the enrichment of beneficial microbial communities containing plant growth-promoting microorganisms and biocontrol agents (Mazoyon et al., 2023), thereby affecting plant health and performance. For instance, canola–pea–barley rotation has been consistently reported to alter the composition of rhizosphere microbial communities, increase yield and oil content, and decrease disease pressure caused by *Leptosphaeria* and *Alternaria* (Town et al., 2023). Although pea has been widely used for crop rotation and as green manure in agricultural systems, the specific role of pea–cucumber rotation in alleviating the obstacles of continuous cucumber cropping and its effects on soil nutrients and microbial communities remain largely unexplored.

In this study, rhizosphere soils were collected from greenhouses subjected to long-term continuous cucumber cropping and from those subjected to pea–cucumber rotation with straw return as green manure, followed by high-throughput sequencing. The composition and diversity of soil bacterial and fungal communities showed significant changes. Among them, bacterial taxa belonging to the genus *Bacillus* exhibited the highest abundance, with a two-fold increase, whereas fungal taxa belonging to *Fusarium* species showed a 4.9-fold reduction after pea–cucumber rotation. In addition, the contents of available nitrogen (AN), available potassium (AK), and available phosphorus (AP) in the soil increased by 1.3-fold after rotation. Correlation analysis revealed significant relationships among soil bacteria, fungi, and nutrients. Specifically, the genus *Bacillus* and available potassium were significantly and negatively correlated with *Fusarium* pathogens, whereas available potassium was significantly and positively correlated with *Bacillus*. Furthermore, the isolated *B. pumilus* and *B. safensis* strains markedly suppressed the growth of cucumber *Fusarium* wilt (FW) pathogens. These findings underscore the importance of investigating the functional mechanisms of pea–cucumber rotation and green manure systems and identifying optimal combinations of beneficial soil bacteria and nutrients for sustainable cucumber production.

## 2 Materials and methods

### 2.1 Rhizosphere soil collection

Rhizosphere soil samples were collected from experimental greenhouse fields cultivated with cucumber or pea seedlings located at Yangzhou University (32°23′35.3″N 119°25′09.1″E, Yangzhou, China). Soil samples were obtained using a randomized complete block design from greenhouses with continuous cucumber cropping and pea–cucumber rotation patterns. Cucumber seeds were germinated overnight in the dark at 28 °C, and the resulting seedlings were grown in a controlled chamber under a 16 h/8 h light/dark cycle at 25 °C/18 °C (day/night), respectively. Cucumber seedlings at the five-true-leaf stage were transplanted into greenhouses under continuous cropping, where they were cultivated through multiple growing seasons until the final harvest. Pea (*Pisum sativum* L.) seeds were sown at a rate of 225 kg/ha with a row spacing of 20 cm. Narrow furrows of optimum depth were opened manually using a hand-operated furrow opener in greenhouses with long-term continuous cucumber cropping. When pea seedlings reached a height of 30–40 cm, they were incorporated into the soil as green manure *via* rotary tillage. Soil samples were then collected from the upper soil layer (10–20 cm) of cucumber monoculture plots (control, X_CK) and pea–cucumber rotation plots (treatment, X_YM) that had been established for more than 2 years, with five replicates per treatment.

### 2.2 DNA extraction, metagenomic sequencing, and analysis

Total genomic DNA was extracted from the different soil samples using a PowerSoil® DNA Isolation Kit (MO BIO Laboratories Inc., Carlsbad, CA, United States). DNA concentration and purity were determined using a NanoDrop 2000 spectrophotometer (Thermo Scientific, Waltham, MA, USA), and DNA integrity was verified by 1% agarose gel electrophoresis. The extracted DNA was then used to analyze the composition and diversity of soil bacterial and fungal communities using high-throughput amplicon sequencing. Primer pairs 341F/806R and ITS1F/ITS2R were used to amplify the hypervariable V3–V4 region of the bacterial 16S rRNA gene and the ITS region of the fungal rRNA gene, respectively (Niem et al., 2020). The purified PCR products were sequenced on an Illumina MiSeq platform (Illumina, San Diego, USA) following the standard operating procedures. Sequence quality was assessed using FastQC to ensure high-quality reads for downstream analyses. The obtained sequences were processed and analyzed using the mothur and quantitative insights into microbial ecology (QIIME) (version 1.9.0) software packages to calculate *α*-diversity, *β*-diversity, and the relative abundance of operational taxonomic units (OTUs), as well as to perform taxonomic assignment and statistical analyses of bacterial and fungal communities (Yue et al., 2024). Finally, the microbial community data were correlated with the measured soil physicochemical properties to reveal potential relationships.

### 2.3 Determination of soil properties

The rhizosphere soil samples were divided into two groups—X_CK and X_YM—with five replicates per group. Soil physicochemical properties, including available nitrogen (AN), available phosphorus (AP), available potassium (AK), and organic matter (OM), were determined as previously described (Li et al., 2020; Yue et al., 2024).

### 2.4 Isolation of rhizosphere *Bacillus* strains

*Bacillus* strains were isolated from the rhizosphere soil samples using a combination of limiting dilution, antibiotic screening, PCR amplification, and sequencing (Das et al., 2025). Briefly, the soil samples were thoroughly homogenized and heat-treated at 65 °C for 2 h to enrich spore-forming bacteria. The treated samples were then suspended and serially diluted in a sterile 0.9% NaCl solution and spread onto nutrient agar plates, followed by incubation at 35 °C. PCR amplification of the bacterial 16S rRNA gene was performed using primer pairs 27F (5′-AGAGTTTGATC(AC)TGGCTCAG-3′) and 1492R (5′-ACGG(CT)TACCTTGTTACGACTT-3′) (Li et al., 2022). The resulting sequences were identified and analyzed using the nucleotide database of the National Center for Biotechnology Information (NCBI).

### 2.5 Effects of *Bacillus* strains on cucumber FW suppression

The isolated *Bacillus* strains, including *B. cereus*, *B. pumilus*, and *B. safensis*, were used to evaluate their antagonistic effects against *Fusarium oxysporum* f. sp. cucumerinum (FOC), the pathogen that causes cucumber FW. The antifungal activity of the three *Bacillus* strains was assessed using a colony diameter inhibition assay. Briefly, 1 mL of bacterial suspension (OD_600_ = 1.0) of each *Bacillus* strain was mixed with 18 mL of potato dextrose agar (PDA) medium, and a 6-mm agar plug of the FOC colony was placed in the center of each plate. The plates were prepared in triplicate. PDA plates containing an equal amount of sterile distilled water instead of bacterial suspension were used as controls. The diameter of the FOC colonies was measured to determine the inhibitory effect of each *Bacillus* strain.

### 2.6 Statistical analysis

The raw sequence data of soil bacterial and fungal communities were deposited in the NCBI database under Bioproject accession numbers PRJNA1312254 and PRJNA1312268, respectively. The experimental data were statistically analyzed using the general linear model procedure in SPSS (version 16.0; IBM Corp., NY, United States). Differences among the groups were assessed using analysis of variance (ANOVA) to determine significant effects.

## 3 Result

### 3.1 Effects of the pea–cucumber crop rotation model on the diversity of bacterial communities

**Figure 1:**
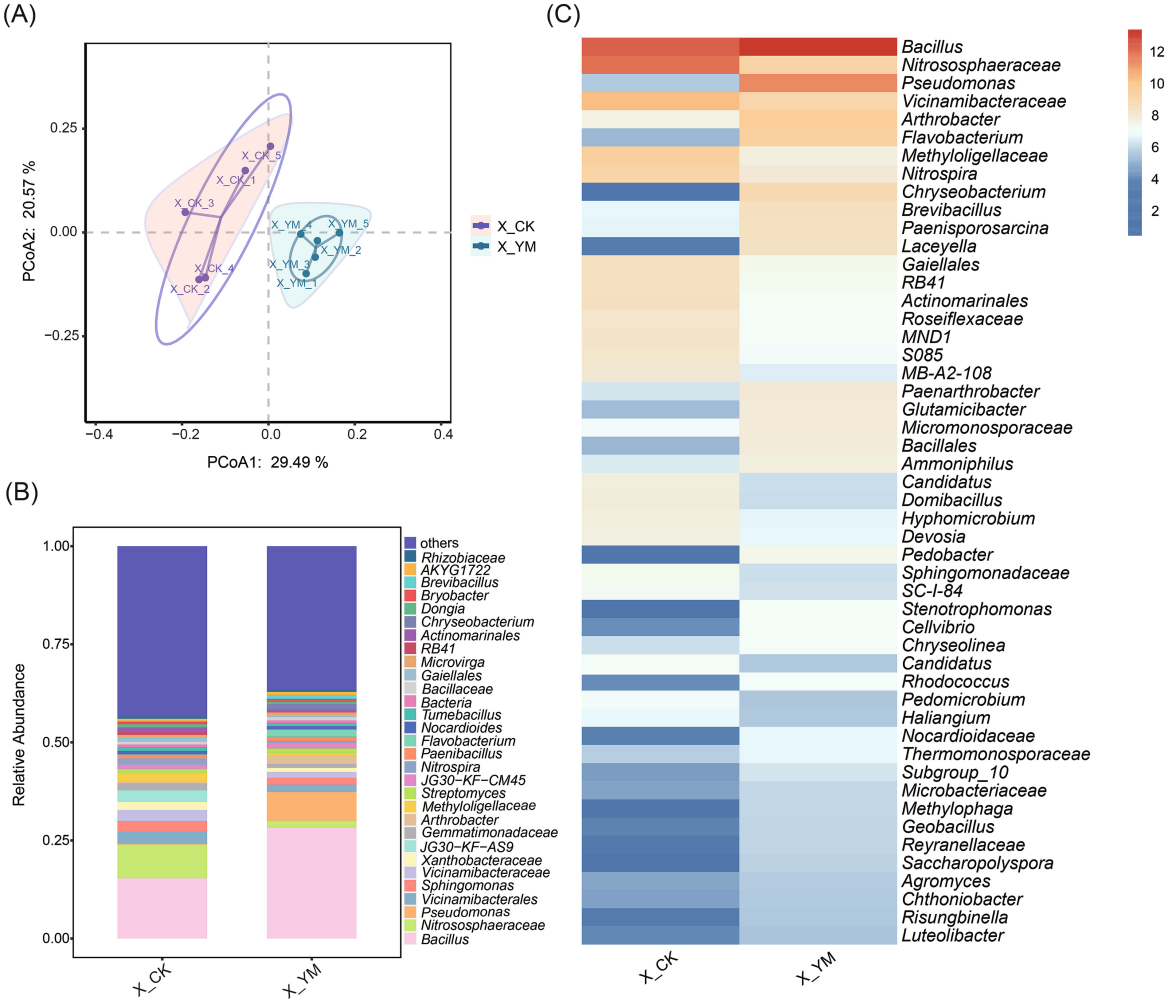
Changes in soil bacterial diversity after pea–cucumber rotation. **(A)** Principal coordinate analysis (PCoA) of bacterial communities under different cropping patterns. **(B)** Relative abundance of bacterial genera. **(C)** Heat map displaying the relative abundance of differential bacterial genera between treatments. X_YM and X_CK represent the samples collected from pea–cucumber rotation and continuous cucumber cropping soils, respectively.

High-throughput sequencing was used to analyze the composition of bacterial communities in soils from the pea–cucumber rotation (treatment, X_YM) and continuous cucumber cropping (control, X_CK) systems. Principal coordinate analysis (PCoA) revealed that the samples from the same treatment clustered closely together, and the five biological replicates from both control and treatment groups were clearly separated (Figure 1A). In addition, the weighted UniFrac distance differed significantly between the two groups (*p* = 0.0024) (Supplementary Figure 1A). The *α*-diversity indices (Richness, Chao1, and Shannon) showed a visible reduction under the rotation treatment, although the differences were not statistically significant (Supplementary Figure 1B).

The number of OTUs was used to characterize bacterial composition, and 409 core OTUs were identified (Supplementary Figure 1C). Taxonomic analysis indicated that the bacterial community exhibited marked diversity, with *Bacillus* species showing a significantly higher relative abundance under the pea–cucumber rotation treatment (Figure 1B). Based on abundance and |log_2_ fold change| > 1 criteria, the top 30 bacterial genera were selected to generate a heat map for differential comparison between the control and rotation treatments (Figure 1C; Supplementary Table 1). Among these, 14 genera showed significantly higher abundance, with the genus *Bacillus* exhibiting the greatest increase, two-fold, under the rotation treatment compared to the control (Figure 1C; Supplementary Figure 1D).

### 3.2 Dynamics of soil fungal communities after pea–cucumber crop rotation

**Figure 2:**
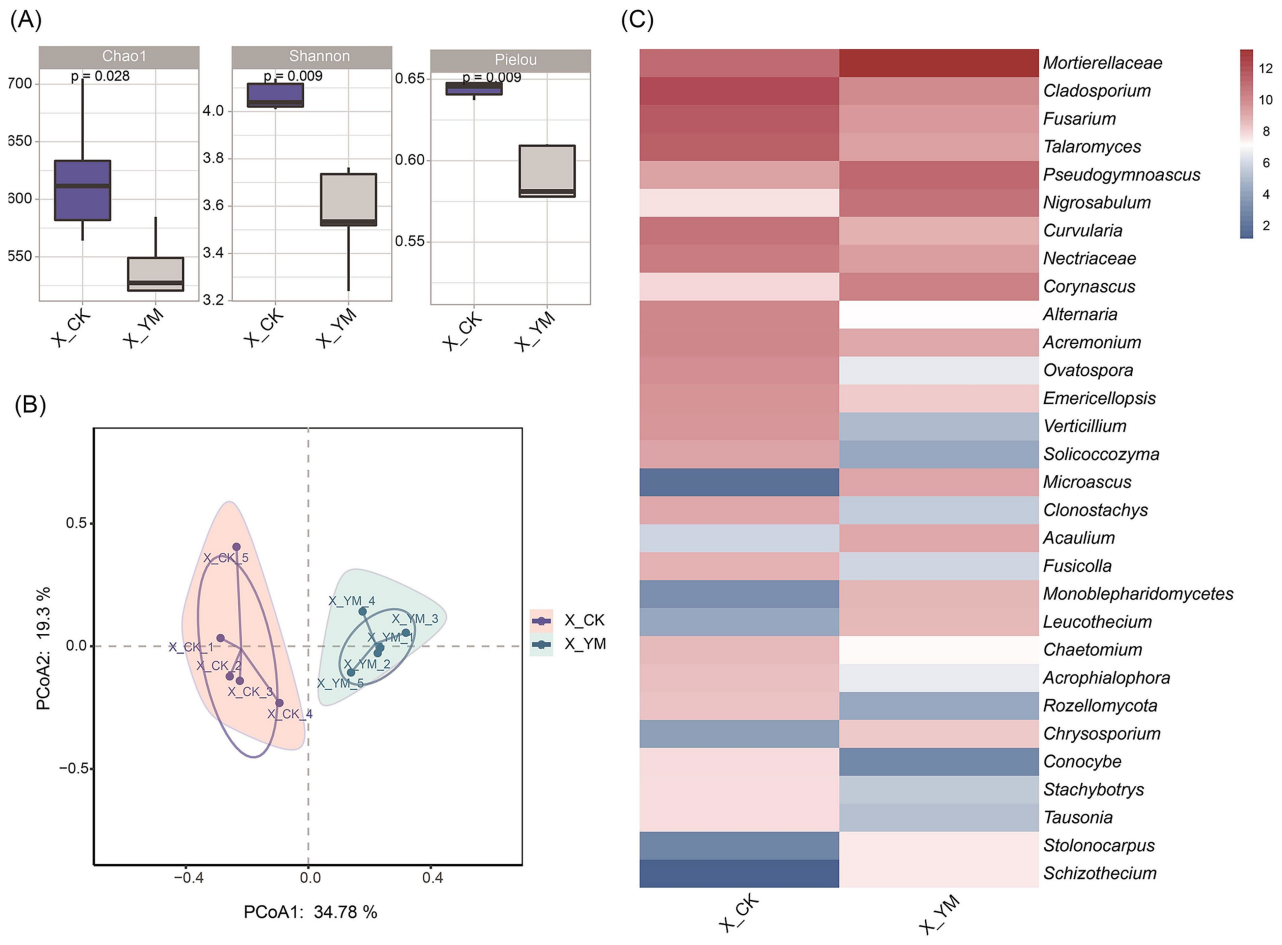
Composition and diversity of fungal communities after pea–cucumber rotation. **(A)** Alpha diversity indices (Chao1, Shannon, and Pielou) of fungal communities. **(B)** Principal coordinate analysis (PCoA) of fungal communities under different cropping patterns. **(C)** Heat map displaying the relative abundance of differential fungal genera between treatments. X_YM and X_CK represent the samples collected from pea–cucumber rotation and continuous cucumber cropping soils, respectively.

To investigate the dynamics of soil fungal communities, *α*-diversity, *β*-diversity, and abundance differences of fungi were analyzed between the pea–cucumber rotation (treatment, X_YM) and continuous cucumber cropping (control, X_CK) soil samples. The α-diversity indices (Chao1, Shannon, and Pielou) showed a significant reduction in the pea–cucumber rotation soil sample (Figure 2A). PCoA demonstrated that the samples from the same treatment clustered closely together (Figure 2B). Taxonomic analysis revealed pronounced diversity in the distribution of fungal species (Supplementary Figure 2). Analysis of differential fungal abundance showed that 19 of the top 30 fungal species decreased significantly in the pea–cucumber rotation soil sample (Figure 2C; Supplementary Table 2), including pathogenic genera such as *Cladosporium*, *Fusarium*, and *Alternaria*, which cause cucumber black spot disease, cucumber *Fusarium* wilt, and cucumber *Alternaria* blight, respectively. In addition, reductions in the abundance of other soil-borne pathogens, including *Didymella*, *Periconia*, and *Verticillium*, were also observed; these pathogens are associated with soil-borne diseases and continuous cropping obstacles (Supplementary Figure 3). These results suggest that the reduction observed in fungal pathogens may be related to changes in bacterial community composition or abundance.

### 3.3 Impact of bacterial communities on fungal diversity and soil-borne pathogens

**Figure 3:**
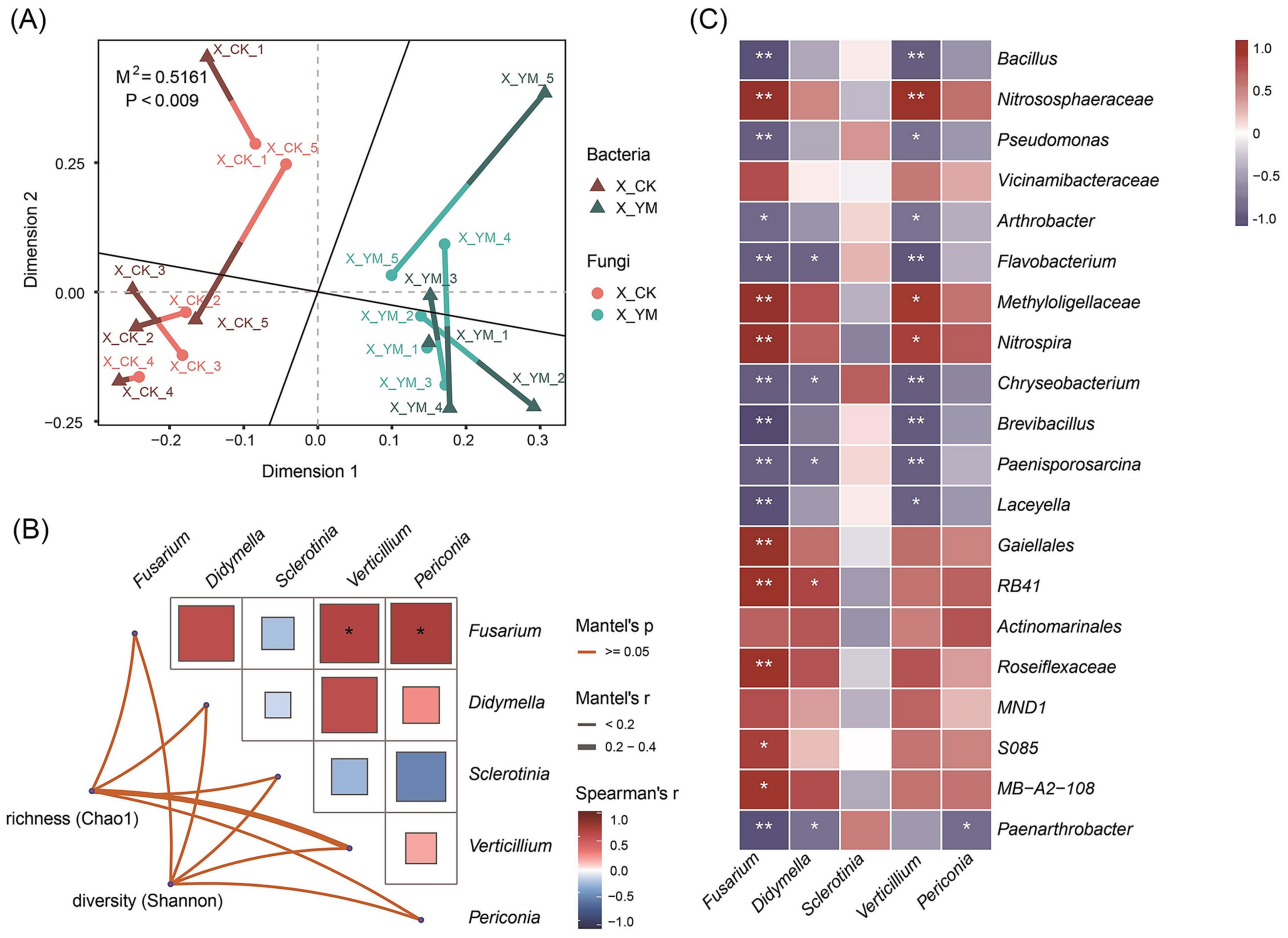
Correlation analysis between soil bacterial diversity and soil-borne fungal pathogens. **(A)** Procrustes analysis of the top 50 differential bacterial and fungal taxa. **(B)** The Mantel test showing correlations between bacterial diversity and soil-borne fungal pathogens. **(C)** Spearman correlation heat map displaying the relative abundance of differential bacterial taxa and soil-borne fungal pathogens. X_YM and X_CK represent the samples collected from pea–cucumber rotation and continuous cucumber cropping soils, respectively. The color scale indicates the correlation strength, with red indicating a positive correlation and blue indicating a negative correlation. “*” and “**” denote significance at *p* < 0.05 and *p* < 0.01, respectively.

Many studies have confirmed that beneficial bacteria play a crucial role in the biological control of pathogens. Both bacterial and fungal communities were significantly altered by the pea–cucumber rotation treatment. To further examine the correlations between bacterial and fungal communities, the top 50 bacterial and fungal taxa with higher abundance and |log_2_ fold change| > 1 were selected for Procrustes analysis to quantify the similarity of spatial patterns between the two groups across samples. The results revealed a significant overall correlation between bacterial and fungal community structures (M^2^ ≈ 0.5161, *p* < 0.009; Figure 3A and Supplementary Table 3). The Mantel test was used to calculate the Mantel correlation coefficient (*r*) and its significance (*p-*value) between the *α*-diversity indices (Chao1 and Shannon) of bacterial communities and the abundance of soil-borne fungal pathogens. The results indicated that the genus *Fusarium* was significantly correlated with *Verticillium* and *Periconia*, although there was no significant correlation between overall bacterial diversity and soil-borne fungal pathogens (Figure 3B). In addition, Spearman correlation analysis was performed between the top 20 bacterial taxa and soil-borne pathogens, and the results were visualized as a heat map. The results showed that nine of the 20 bacterial taxa showed significant negative correlations with *Fusarium* and *Verticillium* pathogens (Figure 3C; Supplementary Table 4), with *Bacillus* species exhibiting the highest relative abundance. These findings suggest that *Bacillus* species may represent a group of beneficial bacteria enriched after pea–cucumber rotation treatment, contributing to the suppression of *Fusarium* pathogens in the soil.

### 3.4 Changes in soil nutrients after pea–cucumber crop rotation

**Figure 4:**
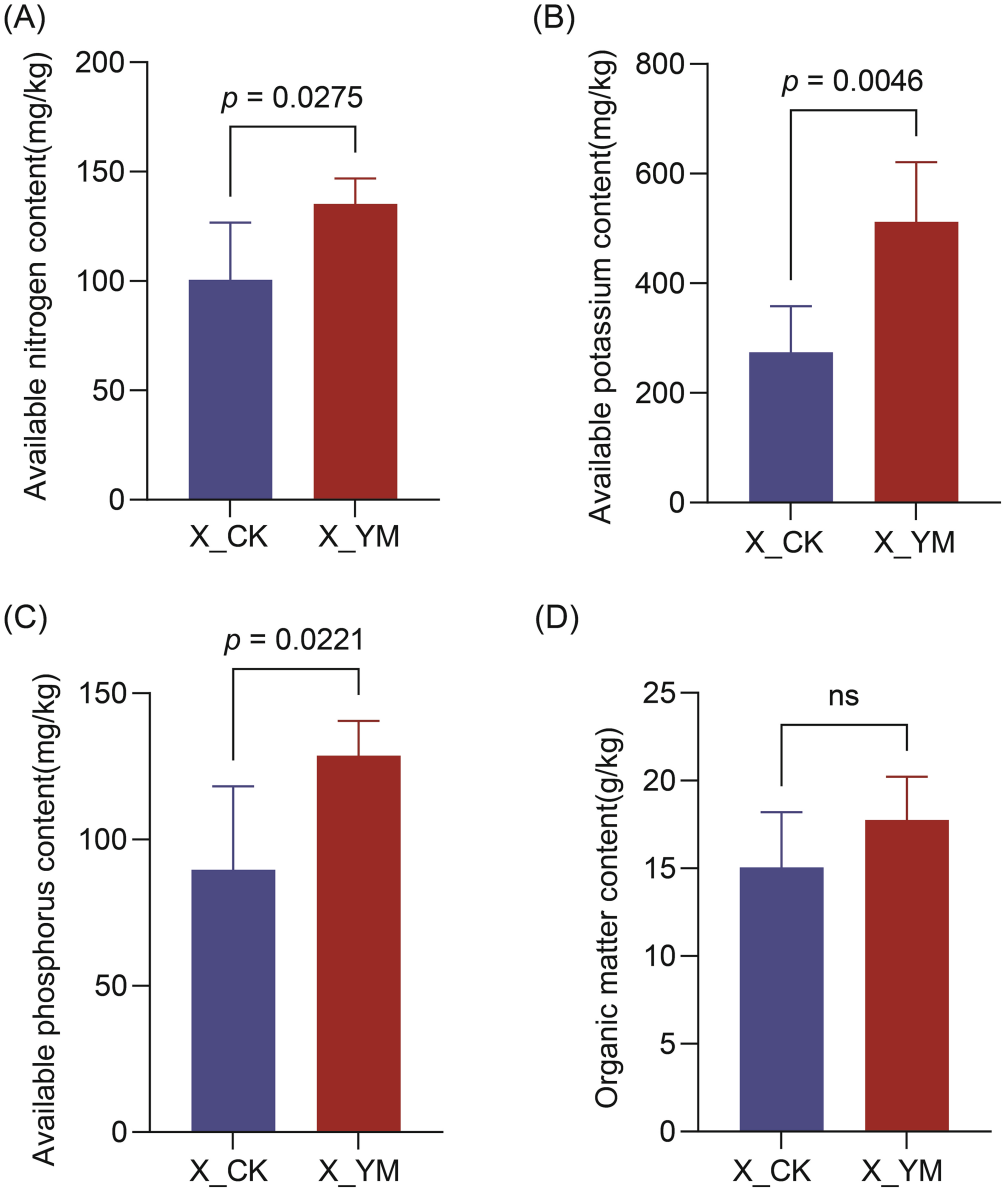
Contents of different soil nutrients: **(A)** available nitrogen, **(B)** available potassium, **(C)** available phosphorus, and **(D)** organic matter. X_YM and X_CK represent the samples collected from pea–cucumber rotation and continuous cucumber cropping soils, respectively. Values are presented as mean ± standard error (SE) of the five replicates. The *p*-values indicate significant differences between treatments.

The basic physicochemical properties of the soil, including AN, AK, AP, and OM, were measured and compared between the pea–cucumber rotation (X_YM) and continuous cucumber cropping (X_CK) treatments. The results showed that the contents of soil AN (135.17 mg/kg), AK (512.55 mg/kg), and AP (128.72 mg/kg) were significantly increased by more than 1.3-fold in the pea–cucumber rotation treatment compared to the continuous cucumber cropping control (Figure 4; Supplementary Table 5). In particular, the contents of soil AK and AP increased by 86.89 and 43.47%, respectively, after the pea–cucumber rotation treatment. However, the OM content did not differ significantly between the X_YM and X_CK groups. These findings indicate that implementing a pea–cucumber rotation system combined with straw return as green manure greatly alters soil nutrient composition, which may contribute to the regulation of soil microbial communities and the alleviation of continuous cropping obstacles.

### 3.5 Correlation analysis between soil nutrients and microbial communities

**Figure 5:**
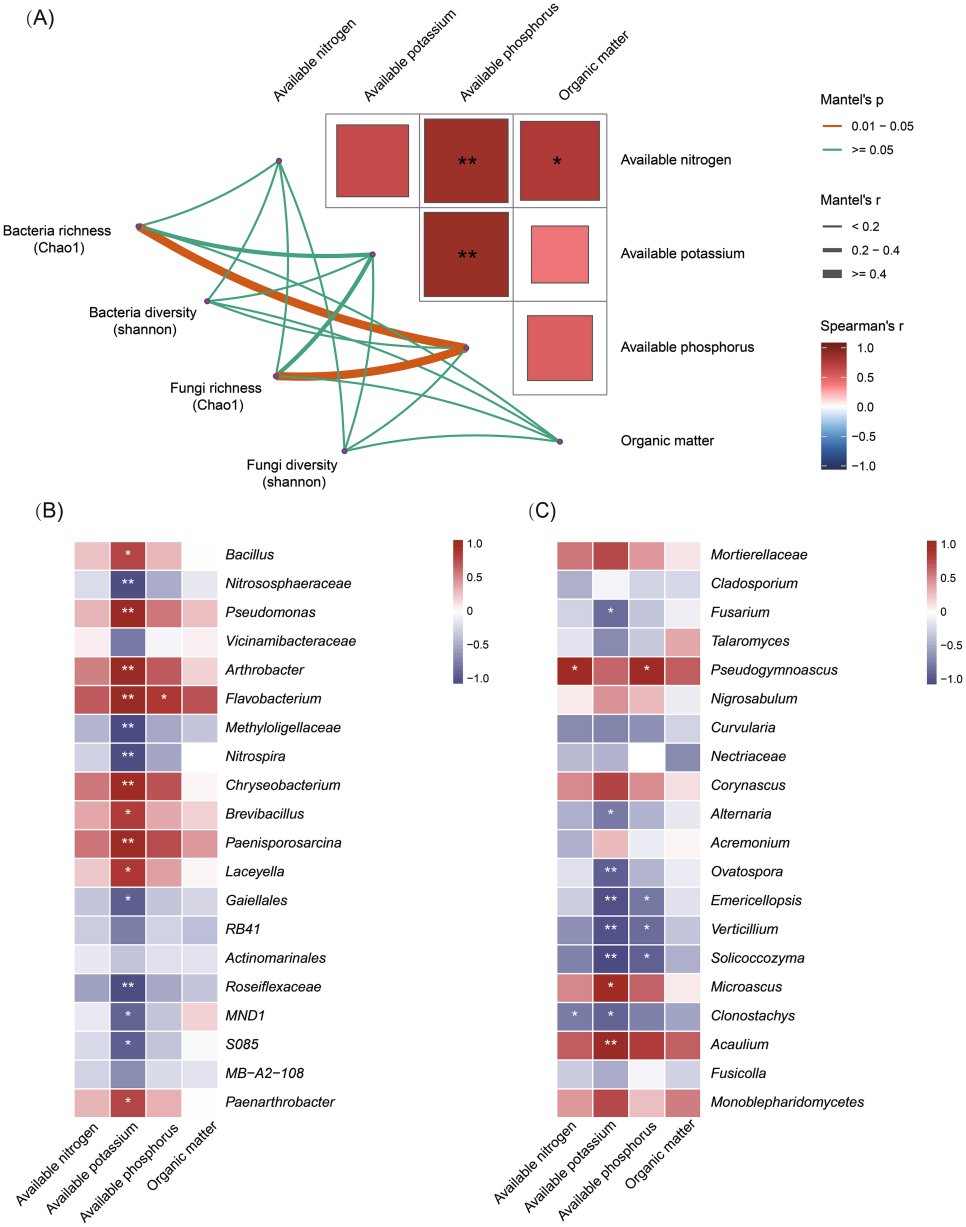
Correlation analysis between soil nutrients and microbial communities. **(A)** The Mantel test showing correlations between soil microbial diversity and nutrient variables. Spearman correlation heat map displaying the relative abundance of differential bacterial **(B)** and fungal taxa **(C)**. The color scale indicates the correlation strength, with red indicating a positive correlation and blue indicating a negative correlation. “*” and “**” denote significance at *p* < 0.05 and *p* < 0.01, respectively.

To explore the relationships between soil nutrients and microbial communities, the Mantel test was performed between the bacterial and fungal diversity indices (Chao1 and Shannon) and the matrix of soil nutrient variables by calculating the coefficient (*r*) and significance (*p*-value). The results showed that AP was significantly correlated with AN and AK (*p* < 0.01), and OM was significantly correlated with AN (*p* < 0.05). In addition, the Chao1 diversity index of both bacterial and fungal communities was significantly correlated with AP (*p* < 0.05) (Figure 5A). Moreover, the top 20 differential bacterial and fungal taxa were selected for correlation analysis with soil nutrient factors. A heat map was generated to visualize significant statistical correlations, providing a basis for interpreting the bacterial and fungal communities and their functional patterns. The results revealed that AK was significantly and positively correlated with nine bacterial taxa, including the genus *Bacillus*, and negatively correlated with seven bacterial taxa (Figure 5B; Supplementary Table 6). AK was also significantly and negatively correlated with seven fungal taxa, including the genera *Fusarium* and *Verticillium* (Figure 5C; Supplementary Table 7). These findings suggest that AK may be an important soil nutrient influencing bacterial and fungal community structures.

In addition, AN was significantly and positively correlated with *Pseudogymnoascus* and negatively correlated with *Clonostachys*. AP was significantly and negatively correlated with *Emericellopsis*, *Verticillium*, and *Solicoccozyma* and positively correlated with *Pseudogymnoascus* (Figure 5C; Supplementary Table 7). Overall, these results indicate that soil nutrient variables strongly and significantly regulate the dynamics of bacterial and fungal communities.

### 3.6 Effects of *Bacillus* strains on cucumber FW suppression

**Figure 6:**
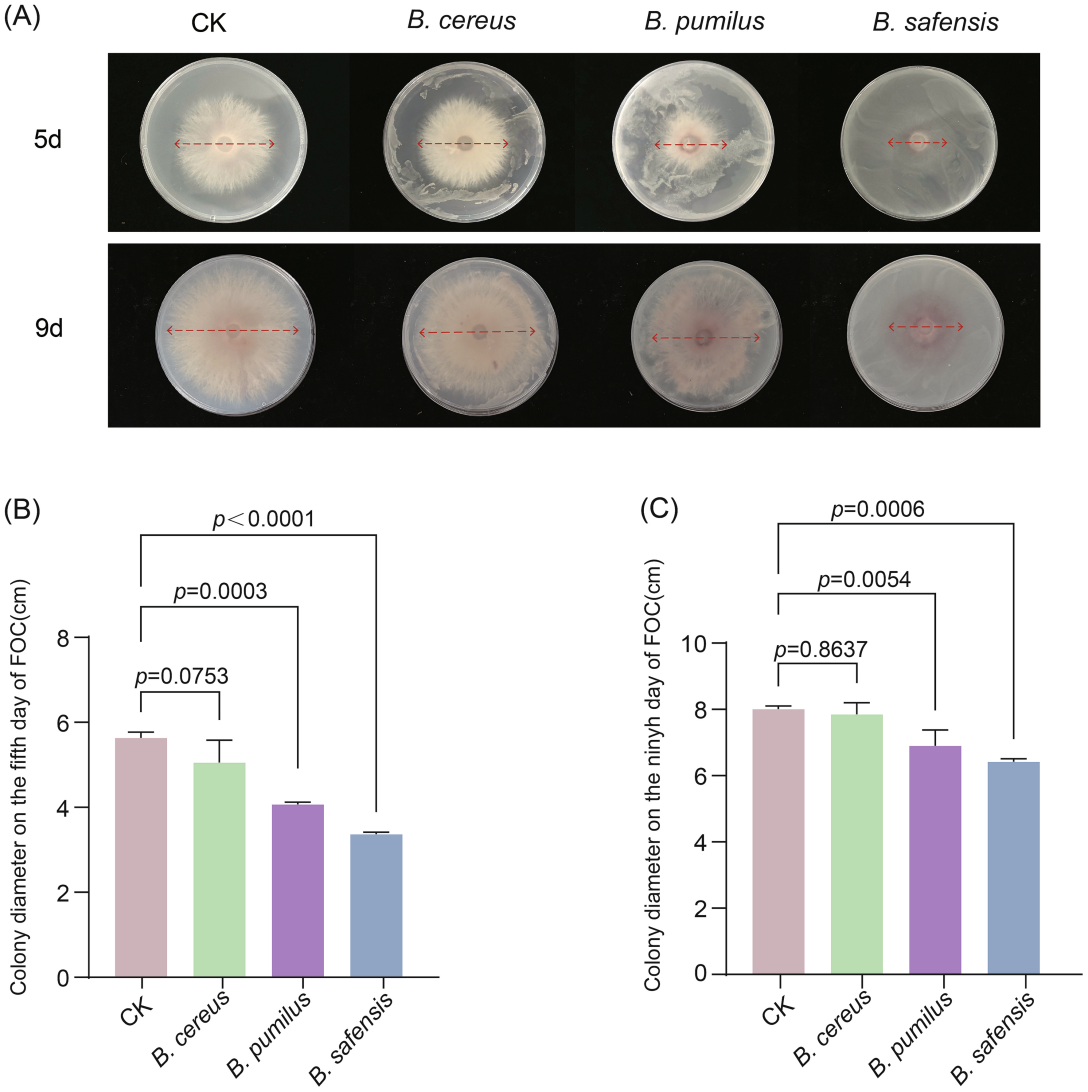
Effects of *Bacillus* strains on FOC suppression. **(A)** Differences in FOC growth on PDA media containing *Bacillus* strains after 5 and 9 days of incubation. Colony diameter of FOC after incubation with *Bacillus* strains for **(B)** 5 days and **(C)** 9 days. Values are presented as mean ± standard error (SE) of the five replicates. The *p*-values indicate significant differences between treatments.

Based on the above analysis, *Bacillus* species exhibited the highest abundance in soil after the pea–cucumber rotation treatment and showed significant negative correlations with *Fusarium* pathogens. Accordingly, *Bacillus* strains were isolated from the soil and evaluated for their ability to suppress FOC, the causal agent of cucumber *Fusarium* wilt. A total of three *Bacillus* strains (*B. cereus*, *B. pumilus*, and *B. safensis*) were isolated and identified using PCR amplification and sequencing (Supplementary Table 8). The antagonistic activity of these strains against FOC was assessed using colony diameter inhibition assays after incubation periods of 3, 5, 7, and 9 days. Compared to the control (FOC grown on medium without *Bacillus* strains), FOC colonies co-cultured with *B. pumilus* and *B. safensis* were significantly suppressed at multiple incubation time points (Figure 6; Supplementary Figure 4). Notably, *B. safensis* significantly suppressed FOC growth, reducing the colony size by 40.15 and 28.33% after 5 and 7 days of incubation, respectively (Figures 6A,B; Supplementary Figure 4B). These findings suggest that *Bacillus* strains exert positive effects on cucumber FW suppression. Therefore, it can be inferred that the combined enrichment of beneficial *Bacillus* species and soil nutrients may contribute to improved soil ecosystem health.

## 4 Discussion

### 4.1 Negative effects of the continuous cucumber cropping system

Long-term continuous cropping often results in soil sickness, characterized by nutrient imbalance, accumulation of soil-borne pathogens, and subsequent declines in crop yield and quality (Banerjee et al., 2019; Wu et al., 2020; Chen et al., 2022). In this study, greenhouse soils under continuous cucumber cultivation (X_CK) exhibited high abundance of multiple harmful fungal pathogens, including the genera *Fusarium*, *Cladosporium*, *Alternaria*, *Didymella*, and *Periconia*, which are known to cause cucumber *Fusarium* wilt, black spot disease, *Alternaria* blight, gummy stem blight, and other diseases (Figure 2; Supplementary Figure 3). These findings highlight the challenges and negative effects associated with continuous cucumber cultivation. Previous studies have also demonstrated that prolonged cucumber monocropping in greenhouses adversely affects cucumber yield and quality, leading to decreased contents of vitamin C, soluble sugars, and proteins in cucumber (Sun et al., 2021). Moreover, with increasing years of continuous cucumber cropping (0, 4, 8, and 12 years), total soil salinity progressively increased, whereas soil pH significantly decreased (Huang et al., 2023). Similarly, an investigation of rhizosphere soils under 1, 3, 5, and 7 years of continuous cucumber cropping in solar greenhouses revealed that a longer cropping duration led to a gradual increase in the relative abundance of *Proteobacteria*, *Chloroflexi*, *Gemmatimonadetes*, *Patescibacteria*, and *Firmicutes*, accompanied by a significant decrease in *Actinobacteria* (Li et al., 2021).

### 4.2 Changes in microbial communities and soil nutrients regulated by the crop rotation model

In diverse agricultural systems, crop rotation can exert long-term effects by improving soil nutrient dynamics and enhancing the abundance of beneficial microorganisms (Wang et al., 2023; Wu J. et al., 2025). In this study, rotation with pea led to pronounced changes in rhizosphere bacterial and fungal communities and soil nutrient levels. In particular, potentially plant-beneficial bacteria (such as *Bacillus* spp.) showed a significant increase in abundance, whereas several pathogenic fungi, including *Fusarium*, *Cladosporium*, and *Alternaria*, were markedly reduced after pea–cucumber rotation (Figures 1, 2). In addition, the contents of soil AN, AK, and AP in the soil increased by more than 1.3-fold, with AK and AP increasing by 86.89 and 43.47%, respectively (Figure 4; Supplementary Table 5). Similar findings have been reported for other cucumber-based rotation patterns that reshape soil microbial communities and alter nutrient composition to suppress pathogen infections. For example, pepper–cucumber rotation promoted the enrichment of two *Pseudarthrobacter oxydans* strains, which markedly reduced root-knot nematode (*Meloidogyne incognita*) infection and diminished the accumulation of *p*-hydroxybenzoic acid in the soil (Tian et al., 2025). Similarly, cress–cucumber rotation greatly increased the relative abundance of potentially beneficial microorganisms, including *Roseiflexus*, *Pseudallescheria* spp., and *Haliangium* spp., thereby suppressing potential pathogenic fungi, such as *Fusarium* and *Monographella* spp., and improving cucumber yield (Gong et al., 2021). In addition, rotations with Indian mustard (*Brassica juncea*) and wild rocket (*Diplotaxis tenuifolia* (L.) DC.) were found to restructure rhizobacterial communities and enhance the populations of potential plant-beneficial microorganisms (such as *Pseudomonas* spp.), which significantly suppressed cucumber *Fusarium* wilt disease (Jin et al., 2019b). Collectively, these findings indicate that crop rotation models positively affect soil microbial ecology by enriching beneficial bacteria, reducing harmful pathogens, and improving nutrient availability, thereby promoting cucumber growth and yield. Nevertheless, the molecular mechanisms underlying these effects and the optimization and broader applicability of the pea–cucumber rotation model warrant further in-depth investigation.

### 4.3 Interactions between microbial communities and soil nutrients

Interactions between soil nutrients and microbial communities play critical roles in modulating soil fertility and health, thereby enhancing the natural resilience of soils against degradation and disease (Goodall et al., 2025). In this study, numerous bacterial taxa and soil physicochemical properties were found to be significantly correlated with pathogenic fungi. In particular, the abundance of *Bacillus* and AK content both increased significantly and were negatively correlated with the relative abundance of *Fusarium* pathogens. Moreover, AK was significantly and positively correlated with the relative abundance of *Bacillus* (Figures 3, 5). Accumulating evidence suggests that interactions between microbial communities and soil nutrients can positively affect plant resistance and productivity. For example, the application of a bio-organic fertilizer composed of organic matter (284 g/kg), total nitrogen (25.8 g/kg), total potassium (14.9 g/kg), total phosphorus (42 g/kg), and *Bacillus amyloliquefaciens* SQR9 (>10^7^ CFU/g) in continuous cucumber cropping systems substantially altered soil microbial communities and physicochemical properties, leading to effective suppression of cucumber *Fusarium* wilt and increased yield (Huang et al., 2017). Furthermore, the diversity of soil microbial communities plays an important role in maintaining soil nutrient balance and ecosystem stability (Shu et al., 2024). Another study demonstrated that soil microbial communities could regulate organic matter dynamics, nutrient cycling—such as organic carbon, total phosphorus, and NH4^+^-N—and the degradation of soil pollutants (Han et al., 2024). Future studies should focus on elucidating the interactive relationships and regulatory networks between soil microbial communities (such as *Bacillus* spp.) and nutrients (such as AK) to improve soil ecosystem health and enhance the suppression of cucumber FW disease.

### 4.4 Functions of *Bacillus* strains in improving cucumber production and disease suppression

In recent years, various beneficial fungi and bacteria have been identified and applied to improve soil health, enhance plant resistance, and promote crop growth (Tzipilevich et al., 2021; Ma Y. et al., 2025; Moreira et al., 2025). For example, two *Paenibacillus polymyxa* strains, NSY50 and WLY78, have been reported to produce antifungal compounds that strongly suppress the growth of *Fusarium oxysporum* and regulate rhizospheric microbial communities, thereby promoting cucumber growth (Shi et al., 2017; Li and Chen, 2019; Du et al., 2022). Similarly, the application of *Trichoderma harzianum* SQR-T037 has been shown to rapidly degrade allelochemicals, significantly suppress *Fusarium* wilt, and alter microbial community composition in continuously cropped cucumber soils (Chen et al., 2011; Chen et al., 2012). In this study, two *Bacillus* strains, *B. pumilus* and *B. safensis*, were identified and exhibited strong suppression effects on FOC growth. In particular, *B. safensis* highly inhibited FOC growth, reducing the colony size by 40.15 and 28.33% after 5 and 7 days of incubation, respectively (Figure 6; Supplementary Figure 4). Previous studies have reported that numerous antagonistic bacteria belonging to the *Bacillus* genus, such as *B. velezensis* NH-1, *B. siamensis* NB92, *B. amyloliquefaciens* FH-1, *B. ayatagriensis* RMG6^T^, and *B. amyloliquefaciens* B2, are widely used to suppress cucumber FW pathogens and promote sustainable agricultural production (Luo et al., 2019; Wang J. et al., 2021; Wang H. et al., 2021; Das et al., 2025; Wang et al., 2025). Moreover, *Bacillus* species can activate beneficial microorganisms and secrete antifungal compounds that enhance disease resistance, thereby promoting the growth and health of cucumber (Wang and Sugiyama, 2024; Wang et al., 2025). Therefore, future studies should focus on exploring optimized combinations of *Bacillus* species with other beneficial microorganisms and identifying the specific antifungal metabolites secreted by *Bacillus* strains. These efforts will provide a more comprehensive understanding of the mechanisms underlying cucumber FW disease suppression and help alleviate the challenges of continuous cropping.

## 5 Conclusion

This study demonstrated that pea–cucumber rotation combined with straw return as green manure enhanced soil nutrient levels and reshaped the composition of complex microbial communities. The intricate correlations and interactions among soil nutrients, bacterial and fungal communities, and soil-borne pathogens were investigated, revealing their collective contribution to cucumber FW disease resistance. Two isolated *Bacillus* strains, *B. pumilus* and *B. safensis*, exhibited strong antagonistic activity against cucumber FW pathogens. Future studies should focus on elucidating the antifungal mechanisms of *B. pumilus* and *B. safensis* and exploring optimized combinations of *Bacillus* species, other beneficial microorganisms, and key soil nutrients to overcome continuous cropping obstacles and improve sustainable cucumber production.

## Data Availability

The datasets presented in this study can be found in online repositories. The names of the repository/repositories and accession number(s) can be found in the article/Supplementary material.

## Supplementary material

Supplementary Figure 1 Structure and diversity analyses of soil bacterial communities.

Supplementary Figure 2 Structure and diversity analyses of soil fungal communities.

Supplementary Figure 3 Relative abundances of soil-borne fungal pathogens.

Supplementary Figure 4 Colony diameter of FOC after incubation with *Bacillus* strains for 3 days and 7 days.

Supplementary Table 1 The abundance of soil bacterial communities, and the top 50 differential bacteria.

Supplementary Table 2 The abundance of soil fungal communities, and the top 50 differential fungi.

Supplementary Table 3 The Procrustes analysis of the top 50 differential bacteria and fungi.

Supplementary Table 4 The significance (*p* value) and coefficient (*r*) value between the soil-borne fungal pathogens and top 20 differential bacteria.

Supplementary Table 5 The content of different soil nutrients in the pea-cucumber rotation (X_YM) and cucumber continuous cropping soils (X_CK).

Supplementary Table 6 The significance (*p* value) and coefficient (*r*) value between soil nutrients and top 20 differential bacteria.

Supplementary Table 7 The significance (*p* value) and coefficient (*r*) value between soil nutrients and top 20 differential fungi.

Supplementary Table 8 The identified information of rhizosphere *Bacillus* sp. strains.
