# Supplementary figures and images for: Pea–cucumber crop rotation suppresses Fusarium pathogens by reshaping soil microbial communities and enhancing nutrient availability
Source: Front Microbiol. 2025 Nov 12;16:1697343. doi: 10.3389/fmicb.2025.1697343 (PMC12647031; doi:10.3389/fmicb.2025.1697343)

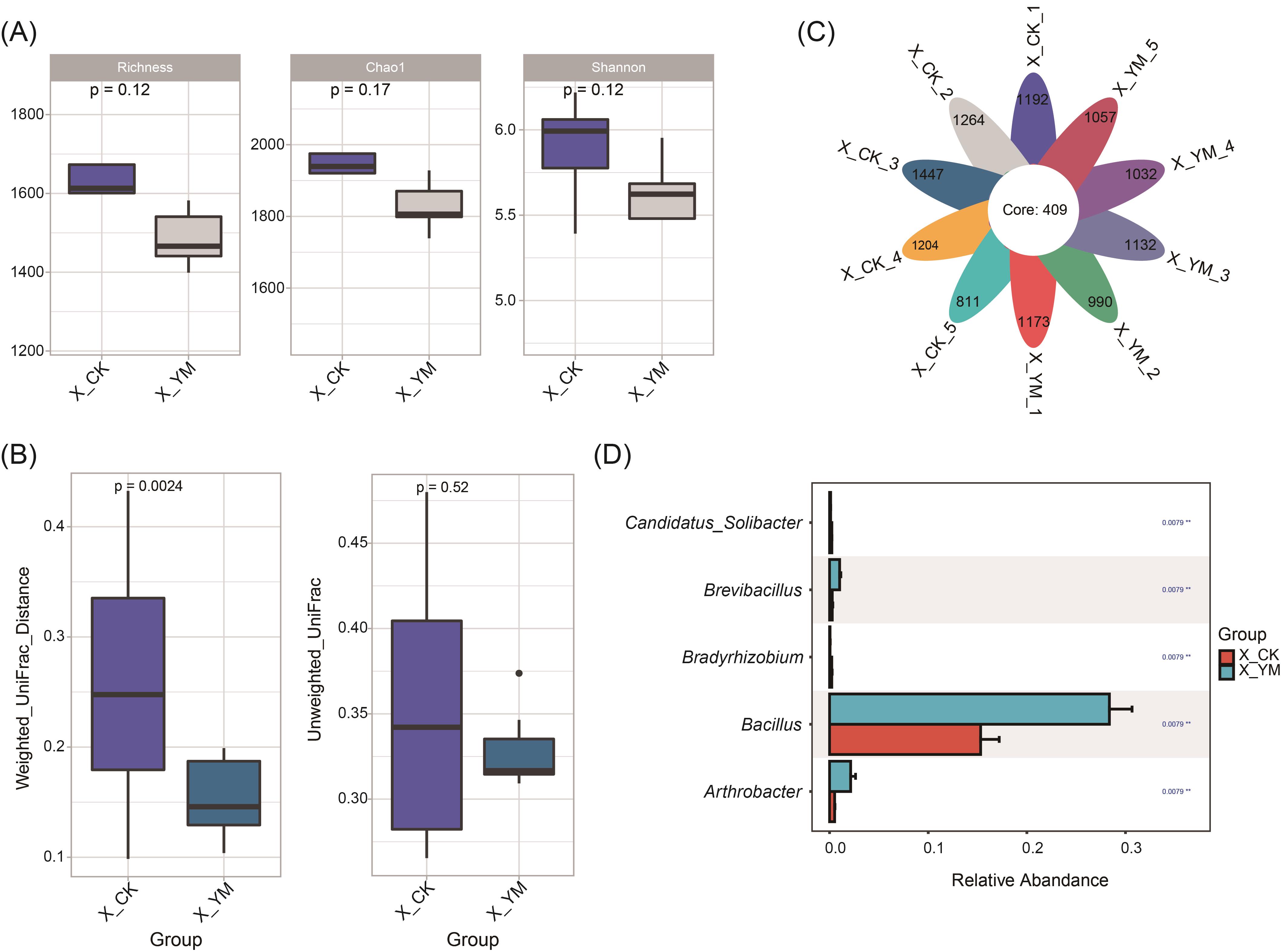

Supplement: Supplementary Figure 1 — Structure and diversity analyses of soil bacterial communities. [file Image_1.JPEG]

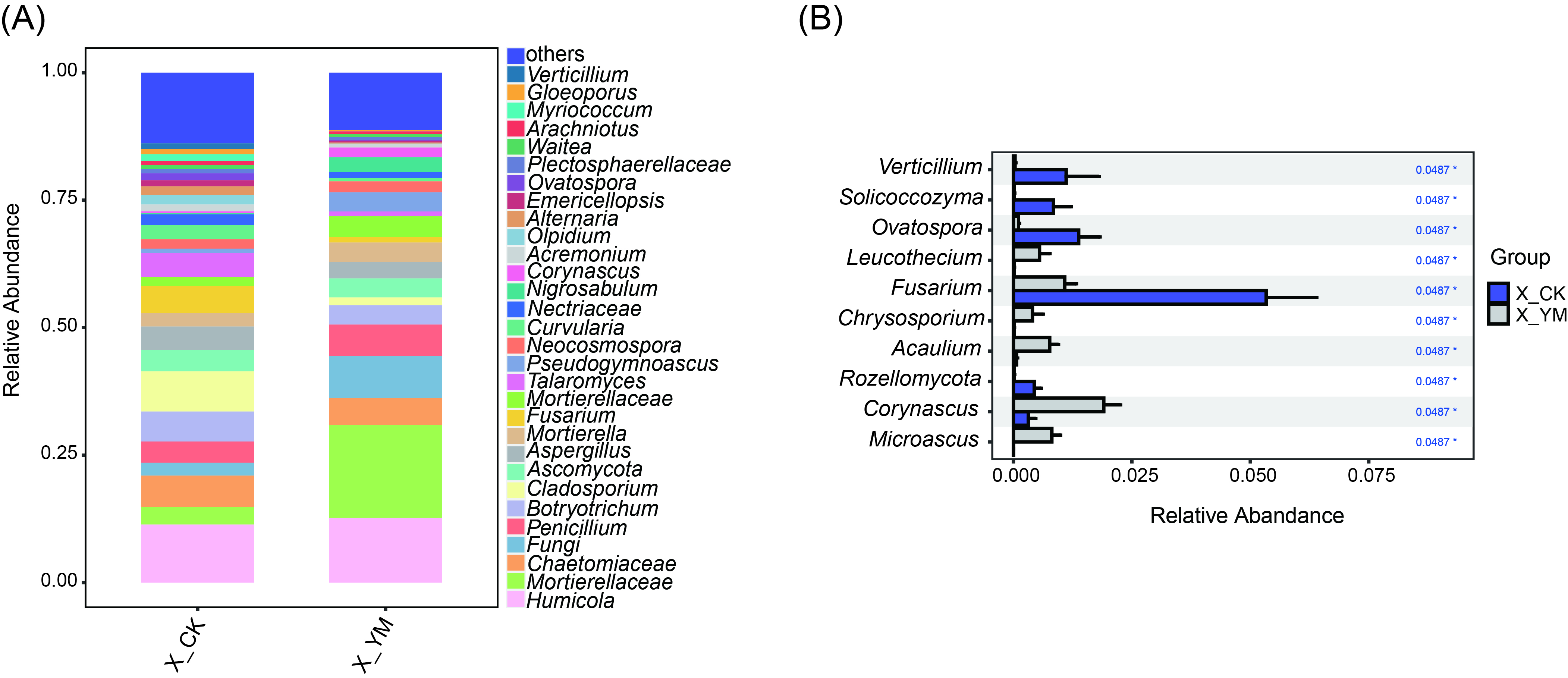

Supplement: Supplementary Figure 2 — Structure and diversity analyses of soil fungal communities. [file Image_2.JPEG]

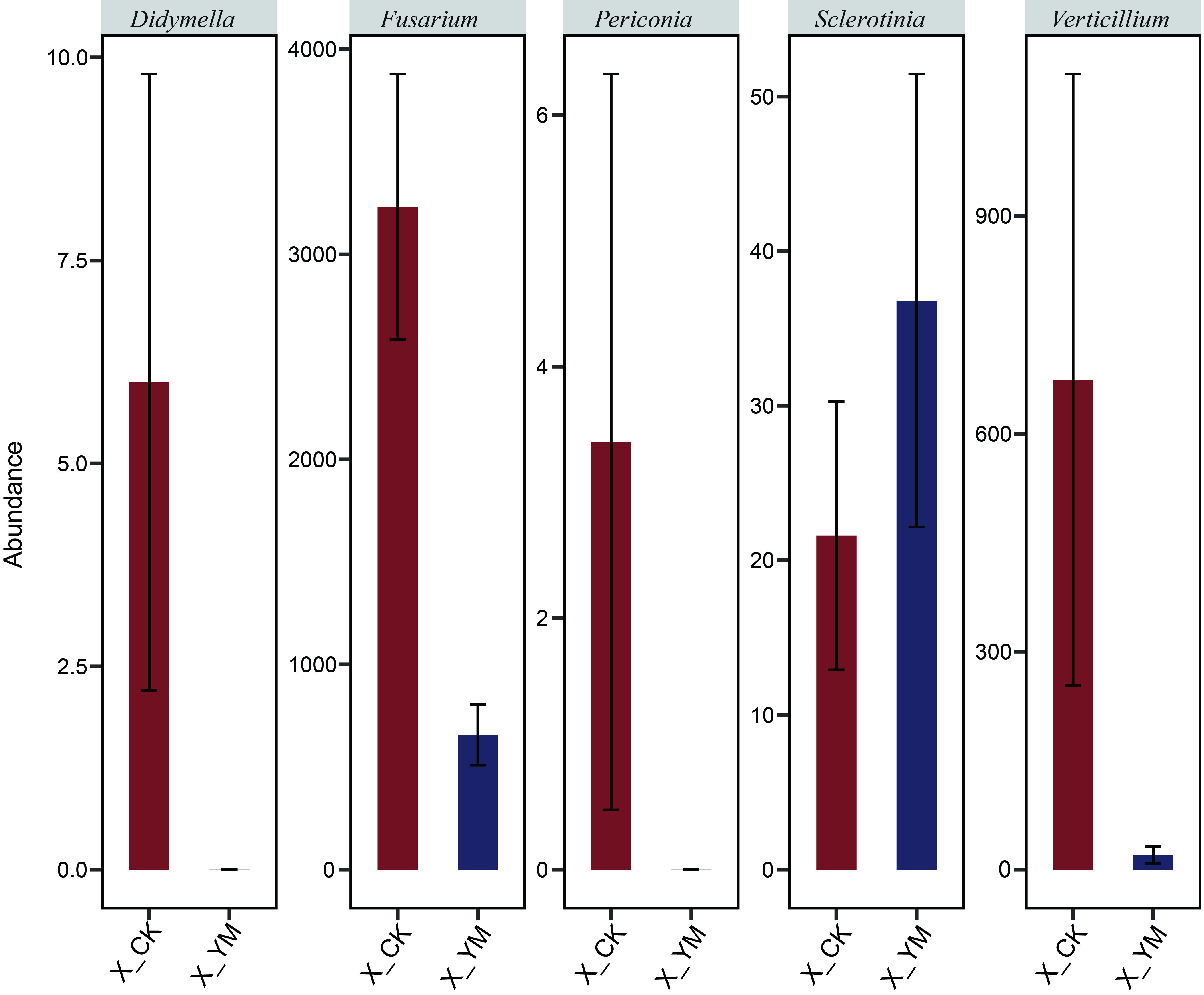

Supplement: Supplementary Figure 3 — Relative abundances of soil-borne fungal pathogens. [file Image_3.JPEG]

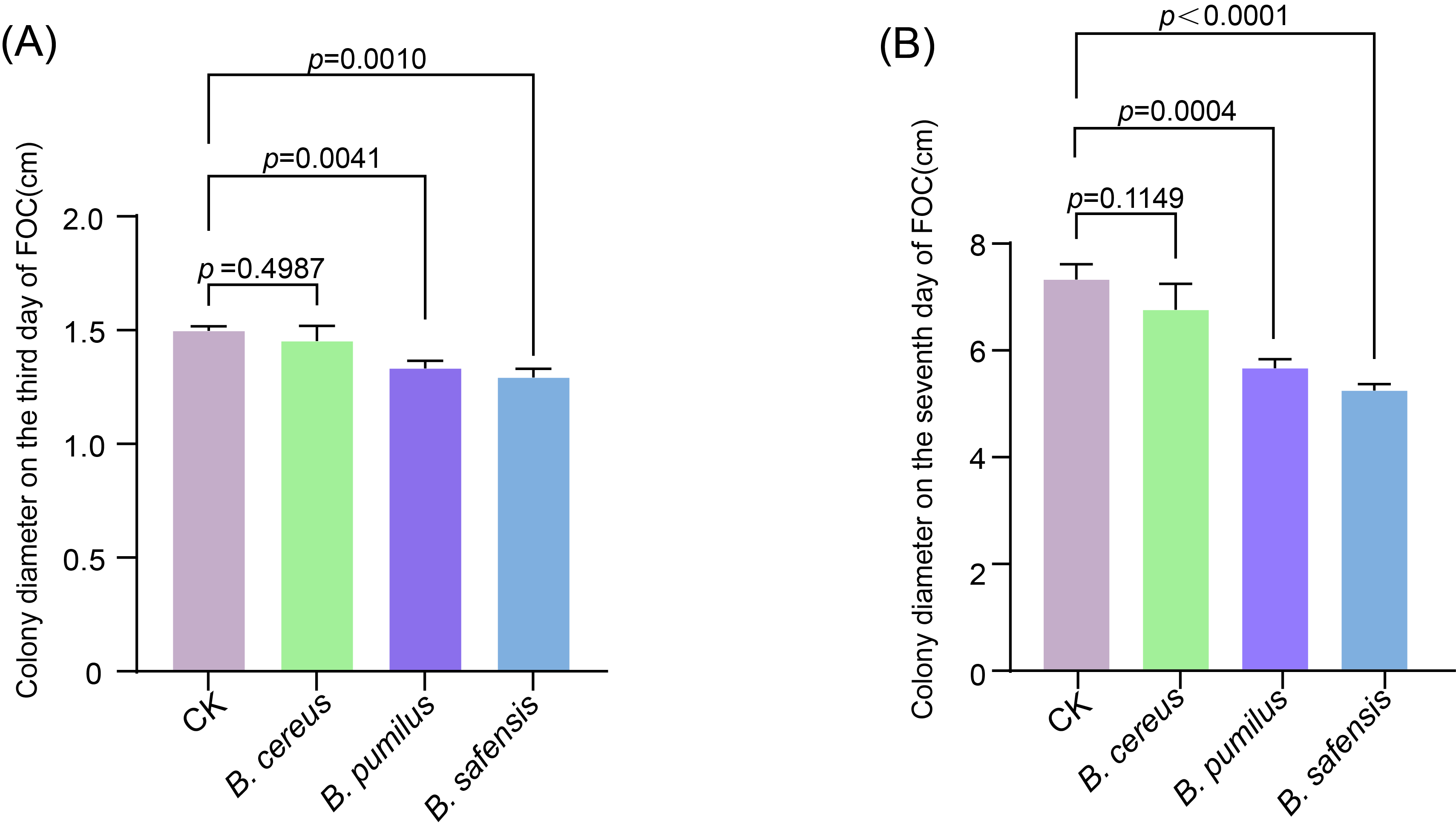

Supplement: Supplementary Figure 4 — Colony diameter of FOC after incubation with Bacillus strains for 3 days and 7 days. [file Image_4.JPEG]
